# Microbiome: Should we diversify from diversity?

**DOI:** 10.1080/19490976.2016.1241933

**Published:** 2016-10-10

**Authors:** Katerina V.-A. Johnson, Philip W. J. Burnet

**Affiliations:** a Department of Experimental Psychology, University of Oxford, Oxford, UK; b Department of Psychiatry, Warneford Hospital, University of Oxford, Oxford, UK

**Keywords:** diversity, ecological theory, gut microbiome, human health, stability

## Abstract

Studies on microbiome diversity are flooding the current literature, yet lessons from ecology clearly demonstrate that diversity is just one factor to consider when analyzing an ecosystem, along with its stability, structure and function. Measures of diversity may be a useful tool for interpreting metagenomic data but the question remains as to how informative they are and what insight they may provide into the state of the microbiome. A study utilizing mathematical modeling to investigate the ecological dynamics of microbial communities has shown that diversity and stability may not always be concomitant. This finding is pertinent to the gut microbiome field, especially since diversity comparisons between healthy and pathological states frequently yield contradictory results. There is a need to broaden our approach to the analysis of microbiome data if we are to better understand this complex ecological community and its role in human health and disease.

With the gut microbiome gaining ever more research attention, data generated via high-throughput sequencing technologies are accumulating faster than our knowledge of how to interpret them. Diversity analyses are frequently applied to microbiome data but there is currently limited understanding of how informative such measures may be in assessing the state of the gut microbial community. Topical research published by Coyte et al.^1^ in *Science* highlights the valuable insight that can be gained by considering the microbiome from an ecological perspective. They present a series of elegant mathematical models, rooted in ecological theory, exploring how numerous factors such as diversity and microbial competition may interact to influence microbiome stability. A premise for their research is the key finding from theoretical ecology that complex multispecies communities are inherently vulnerable to destabilization.^2^ With respect to the human gut microbiome, this offers an intriguing scenario since it maintains a relatively stable state within individuals^3,4^ despite its exceptionally high species diversity.^5^ In their paper, Coyte et al. seek to address this question and use ecological network theory to show that competition between microbial species can help promote microbiome stability in the face of high species diversity. However, they find that microbial cooperation actually tends to reduce stability since a decrease in abundance of one species can have a knock-on effect for its cooperating species, thereby setting the scene for an unstable microbiome.

The authors' findings are relevant to current microbiome research, especially since many studies use diversity as a key measure in their analysis, often assuming that a diverse gut microbiome is a stable and healthy one. However, as demonstrated in their paper, diversity *per se* does not necessarily equate to a stable microbiome since a large number of interacting species tends to have a destabilizing effect. In support of this, several studies have found that the gut microbiome of formula-fed infants is more diverse but less stable compared to breast-fed infants.^6^ Perhaps then we should more carefully consider the use of diversity indices as reliable indicators of microbiome status. Although results of numerous studies do suggest that reduced microbiome diversity may be associated with ill health,^7-9^ this is certainly not always the case,^10-13^ casting doubt on the value of such diversity comparisons between healthy and diseased individuals. For example, a recent study reported that patients suffering from manic depressive disorder had a more diverse gut bacterial community compared to healthy controls.^13^ Though these findings were described as unexpected, this elevated diversity in the depressed individuals may in fact reflect a more unstable gut microbiome. Interestingly, previous sufferers of the disorder who had responded successfully to treatment showed similar gut microbiome diversity measures to the control group. This underlines the importance of knowing both the original state of the microbial community and how it changes during and after disease. Extensive longitudinal studies will further our understanding of the temporal variation of microbiome composition and diversity and its association with various medical conditions.

We must bear in mind that while diversity is a fundamental concept in ecology, it is rather a simple statistic with which to describe the complexity of this microbial ecosystem. Indeed, diversity indices distil ecological data into a single value that takes into account both species richness (number of different species in a community) and evenness (relative abundance of species).^14^ While an increase in either the number of species, or a more even distribution in their abundances, results in a greater diversity score, indices differ in their sensitivity to these two components of richness and evenness.^15^ Discrepancies between common indices of community diversity (e.g. Shannon's or Simpson's Index) have long been recognized in the field of ecology.^15^ Specifically, Shannon's Index is more sensitive to species richness while Simpson's Index is more sensitive to species evenness.^15^ Microbiome studies employing a range of diversity measures reveal that the differences between them can be considerable, influencing the significance of results.^13,16^ This emphasizes the need for a cautious approach when drawing conclusions from any one diversity index. Furthermore, these indices were inherited from macroecology, calling into question their suitability for analyzing microbial communities. Indeed, they lack sensitivity to rare species,^15^ thus underestimating diversity among low-abundance taxa. However, low-abundance organisms typically dominate the composition of microbial communities^17^ and may therefore play a key role in maintaining stability of the gut microbiome. Perhaps studies should incorporate alternative diversity measures, such as the Tail statistic, that has been developed specifically for 16S rRNA sequence data.^17^ This has proved more effective at capturing the diversity among low-abundance species compared to traditional diversity indices.^17^

While diversity measures do encapsulate useful information relating to ecological structure, they ignore crucial factors such as species composition and interactions. Despite the limitations of popular diversity indices, this is certainly not to say that such analyses are redundant. In fact, in many cases microbiome diversity may be positively correlated with the proportion of competitive interactions and so a diverse microbiome may also indicate a stable one. Indeed, the mathematical modeling by Coyte et al. predicts a “wide range of diversities for which this stabilizing effect [of increased competition] dominates the destabilizing effect of increased species numbers.” Notably, traditional hunter-gatherer communities have the most diverse gut microbiomes known.^18,19^ Such high species diversity likely promotes healthy competition among microbial species and weakens cooperative interactions, thereby maintaining stability of the gut community. In comparison, many of the diseases afflicting industrialized populations may stem from depleted microbiome diversity and the lack of certain microbial species due to over-reliance on antibiotics,^20^ our Western diets^21^ and modern cleanliness.^22^

Coyte et al. also suggest that the host may face a trade-off since enhanced microbial cooperation may improve metabolic efficiency, while negatively affecting stability. This may have important implications since large-scale changes in the gut microbiome have been associated with numerous medical problems including obesity.^23^ Such alterations may therefore reflect an unstable state with increased cooperation within the microbial community and thus more effective energy harvesting from the host's diet. However, we currently have little understanding of which gut microbial species interact cooperatively versus competitively and such knowledge would likely require extensive experimental work, as well as the challenge of culturing anaerobic microbes.

Given the growing interest in artificially altering gut microbial flora, the body of ecological theory presented by Coyte et al. is timely. Future experimental work may seek to test their predictions to determine the applicability of their findings to the human gut microbiome. While probiotics seem unable to persist in the gut given the colonization resistance of a healthy intestinal tract,^24,25^ prebiotics may be used to indirectly manipulate microbiome composition.^26^ Prebiotics are indigestible carbohydrates (e.g., fructo-/galacto-oligosaccharides) which promote the growth and/or activity of certain gut bacteria due to their selective fermentation, particularly by *Bifidobacterium* and *Lactobacillus*.^26^ By enabling these bacterial groups to outcompete other species, and inhibiting the growth of potentially pathogenic bacteria such as *Clostridium* and *Salmonella*,^27^ it may be that prebiotic feeding favors persistence of the more stable microbial communities. However, there is currently limited knowledge about the effects of prebiotics on microbiome stability and diversity but this warrants future investigation.^28^ By understanding how prebiotics influence the ecological dynamics of the microbiome, this may provide insight into the mechanisms underlying their reported health benefits.^29,30^

In conclusion, together with the explosion in empirical microbiome studies, where much of the focus is currently based, computational and mathematical models can often provide insight into such complex ecosystems. This is exemplified by Coyte et al.'s paper where the authors use a modeling approach to simplify interactions within the microbial community and thereby identify key principles governing microbiome dynamics. Their findings have notable implications for analysis of gut microbiome data, indicating that diversity may have a more complicated relationship with microbiome health and stability than often considered. Future studies should look beyond the classic diversity indices and seek to develop and apply novel methods for assessing microbiome composition and functioning, for example the use of bacterial co-occurrence networks to understand how the structure of microbial communities may differ between cohorts.^31^ Additionally, multivariate approaches such as canonical correspondence analysis may further our understanding by revealing associations between the gut microbial community and environmental, physiological and genetic variables. Although rarely adopted in human microbiome research,^32^ such methods are frequently implemented in environmental microbiology^33,34^ and have the advantage of being sensitive to rare species.^35^ This technique has also been developed to account for phylogenetic relationships among bacterial taxa and successfully applied to gut microbiome data.^36^ By assembling a toolkit of methods with which to more accurately analyze microbiome data, this may well facilitate future advances in this burgeoning field.
